# Differences in growth-economics of fast vs. slow growing grass species in response to temperature and nitrogen limitation individually, and in combination

**DOI:** 10.1186/s12898-020-00333-3

**Published:** 2020-11-24

**Authors:** Claudia Colesie, Zsofia Reka Stangl, Vaughan Hurry

**Affiliations:** 1 Edinburgh Global Change Institute, School of GeoSciences, University of Edinburgh, Alexander Crum Brown Road, Edinburgh, UK; 2 Department of Forest Ecology and Management, Swedish University of Agricultural Sciences, Umeå, Sweden; 3 Umeå Plant Science Centre (UPSC), Department of Forest Genetics and Plant Physiology, Swedish University of Agricultural Sciences, Umeå, Sweden

**Keywords:** Plant trait coordination, Stress physiology, Nutrient availability, Invasive species, Functional type, Ecophysiology, Carbon, Strategy, Root

## Abstract

**Background:**

Fast growing invasive alien species are highly efficient with little investment in their tissues. They often outcompete slower growing species with severe consequences for diversity and community composition. The plant economics trait-based approach provides a theoretical framework, allowing the classification of plants with different performance characteristics. However, in multifaceted background, this approach needs testing. The evaluation and prediction of plant performance outcomes in ecologically relevant settings is among the most pressing topics to understand and predict ecosystem functioning, especially in a quickly changing environment. Temperature and nutrient availability are major components of the global environmental change and this study examines the response of growth economic traits, photosynthesis and respiration to such changes for an invasive fast-growing (*Bromus hordaceus*) and a slow-growing perennial (*Bromus erectus*) grass species.

**Results:**

The fully controlled growth chamber experiment simulated temperature—and changes in nitrogen availability individually and in combination. We therefore provide maximum control and monitoring of growth responses allowing general growth trait response patterns to be tested. Under optimal nitrogen availability the slow growing *B. erectus* was better able to handle the lower temperatures (7 °C) whilst both species had problems at higher temperatures (30 °C). Stresses produced by a combination of heat and nutrient availability were identified to be less limiting for the slow growing species but the combination of chilling with low nutrient availability was most detrimental to both species.

**Conclusions:**

For the fast-growing invader *B. hordeaceus* a reduction of nitrogen availability in combination with a temperature increase, leads to limited growth performance in comparison to the slow-growing perennial species *B.erectus* and this may explain why nutrient-rich habitats often experience more invasion than resource-poor habitats.

## Background

The spread of fast-growing invasive alien species is one of the major threats to habitats and their species diversity with implications for plant community assembly in future climate change scenarios. Invasive species may succeed even in low-resource environments by employing resource conservation traits such as high resource-use efficiency [18] and they are typically species located on the ‘fast’ end of the productivity‐persistence trade‐off axes [13]. The ‘plant economics spectrum’ concept provides the theoretical framework to arrange plant species from the ‘fast’ end of the productivity‐persistence trade‐off axis to taxa with ‘slow’, conservative life traits. It integrates across leaves, stems and roots and is a key feature helping to explain individual ecological traits, community assembly processes and the functioning of ecosystems [48]. According to Reich [48], a fast or a slow growth strategy each requires a particular set of leaf, root and stem traits. Plants with a slow growth strategy will have low respiration rates, low nutrient concentrations, dense tissues, with low water movement and loss capacities across all plant tissues. In contrast, fast-growing species are highly efficient in transporting water, in acquiring and using nutrients and in fixing carbon, but invest less in their tissues (whether root, stem, or leaf). The plant individual performance results from the coordinated operation of many processes, such as nutrient uptake, organ turnover or photosynthesis, thus a prediction requires a certain set of traits. A plants economy is determined by its handling/usage of three key resources: carbon, water, and mineral nutrients, and the most critical functional and eco-physiological traits relevant to these.

Functional traits encapsulate the relative and overall constitutive adaptation of plants by revealing the strategies developed under evolutive forces. Therefore, functional traits inform on the overall level of environmental stress in each environment. The most prominent functional trait relevant to the plant’s carbon economics is the specific leaf area (SLA, defined as the amount of leaf area per unit leaf weight). SLA is widely used as a proxy to predict a plants position on the resource use axis [60] and can be considered as the prime factor determining interspecific variation in relative growth rate (RGR, [28]. By definition, leaves with a lower SLA are denser (greater mass per volume) or thicker [46] and tend to invest more in structural leaf defences [9]. The reciprocal of SLA, the leaf mass per area (LMA) is also frequently used [28] as an indicator of plant function [16] and to position a species along an axis based on resources acquisition. The root to shoot ratio (R:S) is a measure of allocation of biomass to roots in relation to aboveground biomass, and can be interpreted according to the “optimal resource partitioning strategy”.

Fast growing species are characterized by having low LMA and high SLA and vice versa for slow growers. For evaluation of a plants nutrient economy, functional traits like nitrogen and phosphorus content, the carbon to nitrogen ratio (C:N) ratio and the nitrogen use efficiency (NUE) are widely used. Nitrogen concentrations are a common leaf and root trait syndrome that links traits to effects on whole plant processes [55]. The C:N ratio of an organ is often regarded as a convenient indicator of growth and quality, and can also be considered as a good indicator of secondary compound concentrations in all plant organs. The NUE (increase in dry weight per unit of nitrogen) describes the efficiency of carbon incorporation into biomass [30].

Other than functional traits, ecophysiological traits condense acclimative processes in an individual plant and they can account for variations in flows of material and energy. The most prominent ecophysiological traits relevant to the plant’s carbon economics are respiration (R) and net photosynthesis (NP, [48]). Products from photosynthesis account for approximately 90% of a plant`s dry weight, therefore the photosynthetic properties of a plant are the basis to understand any variation in growth. However, the daily carbon budget of a plant is also strongly influenced by respiration because approximately 50% of the fixed carbon is respired [44]. Respiration takes places in all plant organs and is therefore very important when whole plant carbon economics are to be understood.

The major outcome of the plant economics trait-based approach is to evaluate performance outcomes in ecologically relevant settings. Many studies exist that test the variability of these traits in response to changes of one parameter, such as irradiance [4, 33], nutrient availability [1, 10, 54], water availability [35–37], and temperature/climate [41, 62]. Under natural conditions, plants are often exposed to complex stresses from several of these resources and the impact of combined effects has been examined under simultaneously varying nutrient and light availabilities [4, 22, 49] as well as nutrient and drought stress [51]. The response of plants to combinations of two or more stressors is unique and cannot be directly extrapolated from the response of plants to each stressor applied individually [53]. The simultaneous occurrence of different stressors results in a high degree of complexity in plant responses because the responses are largely controlled by different, and sometimes opposing, signalling pathways that may interact, both positively and negatively [53]. The question remains open whether the categorisation, implemented by the plant economics spectrum approach, remains valid when individuals of one species are exposed to different and combined effects of stress [11]. For example, it is shown that single-factor studies could be inadequate to forecast plant responses in a climate change scenario [38].

In the climate change context especially, stresses produced by a combination of temperature (both chilling and heat) and nutrient availability were identified as a white spot on the plants stress response matrix [53]. Consequently, we aim to help fill this knowledge gap, by testing the generality of trait relationships and analysing how shifts in temperature and nutrient stoichiometry influence plant functional and ecophysiological traits. The traits we study are considered as ‘hard’ traits, with a direct functional role such as carbon fixation, leaf instantaneous photosynthetic rate, nutrient uptake [21, 31] or SLA [46]. We test a fast-growing, invasive, annual C_3_ grass and a slow-growing perennial species. To differentiate the temperature and nutrient effects, we chose an experimental approach (aeroponic growth chambers) that allows maximum control and monitoring of conditions. We formulate three hypotheses in which trait based plant economics strategies are evaluated against changes in (i) nutrient availability and (ii) temperature individually, and in combination (iii).

(i) Nitrogen limitation will limit growth performance independent of growth strategy but via different routes. While slow growing species have evolved functional traits resulting in a more conservative life strategy that allows growth in low nutrient conditions, fast growing, invasive species will employ resource conservative ecophysiological traits in response to nutrient shortage.
(ii) Temperature affects the plant’s energy balance and metabolic rate. As a response, fast growing annual and slow growing perennial species will respond with similar changes in their ecophysiological trait coordination.
(iii) The individual effects of nutrient or temperature stress are additive when applied in combination. Potential benefits of a more conservative life strategy in slow-growing plants through functional traits vanish when ecophysiological trait coordination is needed as well.

## Results

Because of the multivariate nature and the various interactions, the results are structured to focus on the comparison between the species. Each parameter will be presented separately starting with the comparison of free access nitrogen (FA) for both species against temperature, followed by the low access nitrogen (LA) for both species against temperature.

### Proof of concept

**Fig. 1:**
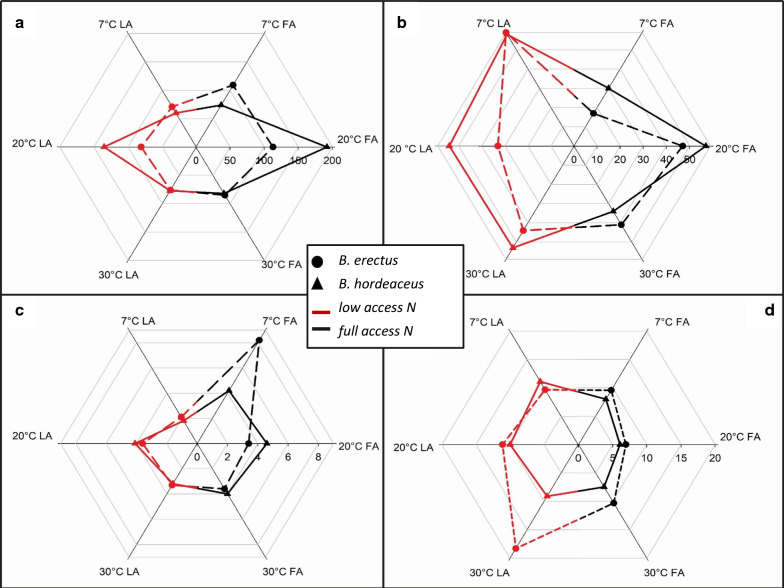
Growth economic traits. **a** Relative growth rate (RGR), **b** Specific leaf area (SLA), **c** Net assimilation rate (NAR) and **d** Carbon to nitrogen ration (C:N ratio)

For both species, RGR and carbon gain were highest when plants were grown at 20 °C with free access nitrogen (Fig. 1a). The LMA values were within the expected range for these species ((52 ± 16 g/m^2^ for *B. erectus* vs. 10 ± 2 g/m^2^ for *B. hordeaceus* [42, 57].

**Fig. 2:**
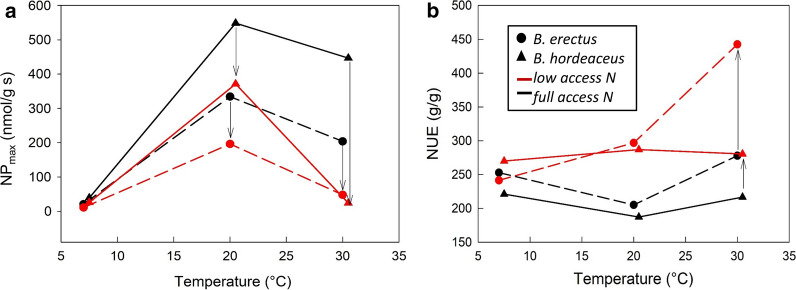
Carbon uptake and nitrogen use efficiency. **a** Maximum carbon uptake rates (NP_max_), **b** Nitrogen use efficiency (NUE). Arrows indicate changes with changes in nutrient availability

At 20 °C, the fast-growth life strategy was validated for *B. hordeaceus* because RGR and NP_max_ were almost twice as high as for *B. erectus* (Figs. 1, 2), C:N ratio was very low, and LMA significantly lower than for *B.erectus* (T-test: t = 5.6, df = 8, p < 0.005). The more conservative life traits were shown by *B. erectus* supporting a slow-growth strategy in this species.

Both species showed the expected suite of characters for their particular growth strategy when grown at 20 °C, FA (Table 1). *B. hordeaceus* had higher RGR, SLA, NP_max_, and lower NUE, C:N and R:S ratios than *B.erectus*, the slow growing species.

**Table 1:** Growth parameters

Parameter	N-treatment	Temp (°C)	*Bromus erectus*	*Bromus hordeaceus*	Test statistics		
					t	df	p
RGR (mg/gd)	FA	7	109	74			
		20	113	192			
		30	85	82			
	LA	7	71	60			
		20	81	135			
		30	76	77			
NAR (g /m^2^d)	FA	7	8.2	4.2			
		20	3.4	4.6			
		30	3.6	4.0			
	LA	7	2.1	1.8			
		20	3.6	4.1			
		30	3.3	3.2			
SLA (m^2^/kg)	FA	7	17 ± 2.3	30 ± 8.1	9.2	8	*0.00*
		20	47 ± 5.3	57 ± 9.3	2.6	8	*0.02*
		30	41 ± 27.5	34 ± 19.0	0.8	8	0.21
	LA	7	59 ± 5.4	58 ± 9.7	6.8	8	*0.00*
		20	33 ± 6.4	54 ± 3.7	4.8	8	*0.00*
		30	44 ± 12.3	53 ± 15.2	1.0	9	0.17
R:S ratio	FA	7	0.52 ± 0.04	0.63 ± 0.06	0.9	12	0.18
		20	0.42 ± 0.03	0.39 ± 0.02	0.1	12	0.46
		30	0.40 ± 0.02	0.37 ± 0.02	0.7	12	0.26
	LA	7	0.46 ± 0.03	0.63 ± 0.06	1.4	12	0.08
		20	0.47 ± 0.04	0.56 ± 0.05	0.8	12	0.22
		30	0.84 ± 0.19	0.83 ± 0.14	0.1	12	0.47
NUE (g/g)	FA	7	252.7 ± 13.8	221.1 ± 17.9	3.1	8	*0.01*
		20	205.1 ± 8.4	187.2 ± 7.2	3.6	8	*0.01*
		30	277.7 ± 19.8	216.6 ± 18.8	5.0	8	*0.00*
	LA	7	241.4 ± 12.8	270.0 ± 31.7	1.9	8	*0.05*
		20	296.7 ± 22.1	286.9 ± 7.7	0.9	8	0.18
		30	442.5 ± 103.6	208.4 ± 15.7	3.4	8	*0.00*

Mean relative growth rate (RGR, mg/g d), net assimilation rate (NAR, g/m^−2^ d) and specific leaf area (SLA, m^2^/kg), root to shoot ratio and nitrogen use efficiency NUE (g/ g) of the fast-growing *Bromus hordeaceus* and the slow-growing *Bromus erectus*, when grown at 7 °C, 20 °C, or 30 °C, with free access (FA) or 50% access (LA) nitrogen

SLA, R:S ratio and NUE values are the average of 5 plants sampled with a fully expanded third leaf (± standard deviation). Test statistics show results from T-tests between the species

### Relative growth rate

For both species, RGR were highest when plants were grown at 20° with FA conditions (Fig. 1a) but there were species-specific responses at other temperatures. The slow-growing *B. erectus* maintained RGR at a high level at 7 °C and around 20% lower at 30 °C whilst the fast-growing *B. hordeaceus,* showed a strong reduction (around 45%) at both 7 °C and 30 °C so that it had a very clear maximum at 20 °C (Table 1).

Under LA conditions, the slow-growing *B. erectus* had similar RGR at all three treatment temperatures at around 65% of FA at 20 °C. In contrast, the fast-growing *B. hordeaceus* again showed a maximum at 20 °C, although this was about 70% of the rate at FA. RGR at 7 °C and 30 °C were almost identical to rates for the slow-growing *B. erectus* at those temperatures.

### Specific leaf area

For both species SLA was maximal under FA conditions at 20 °C which was significantly higher for the fast-growing *B. hordeaceus* (p = 0.016; df = 8; t = 2.58; Fig. 1b). *B. hordeaceus* had a similar SLA at 7 °C and 30 °C, which was about 55% of the maximum. The slow-growing *B. erectus* showed a similar pattern, but SLA was much more reduced at 7 °C than at 30 °C, 36% versus 87% of maximum (Table 1).

Under LA conditions the response patterns varied between the species. The slow-growing *B. erectus* showed a reverse pattern to FA conditions with lowest SLA at 20 °C. SLA was very much higher at 7 °C and 350% higher than in the FA treatment;, and a similar level at 30 °C as under FA conditions. In complete contrast, the fast-growing *B. hordeaceus* had an almost stable SLA with a small and steady decline (Table 1).

### Net assimilation rate

In the FA treatment, the slow-growing *B. erectus* had almost identical net assimilation rate (NAR) at 20 °C and 30 °C. At 7 °C NAR almost doubled (Fig. 1c). Rates for the fast-growing *B. hordeaceus* were similar at all three temperatures and, in general, about 30% higher than for *B. erectus*.

Under LA conditions, there was little difference between the two species in pattern and response to temperature (Fig. 1c). Both species had their highest NAR at 20 °C and lower and similar values at 7 °C and 30 °C.

### Root: shoot ratio

*B. erectus* and *B. hordeaceus* showed a similar response pattern in the FA treatment. The R:S ratio was declining with increasing temperature from 7 °C to 20 °C (Table 1; F_1,73_ = 3.13; p = 0.05), remaining constant at 30 °C. Under LA conditions, the R:S ratio was significantly increased in both species compared to FA conditions (Table 1; F_1,72_ = 11.59; p = 0.002). In response to the combination of warming (30 °C) and low nitrogen availability the R:S ratio was higher for both species but more so, almost doubled, in the slow-growing *B. erectus* (Table 1).

### C:N ratio

Under FA conditions, both species had their lowest ratios at 20 °C and the ratio was increased at higher and lower temperatures (Table 2), with this effect being greater in the slow-growing *B. erectus* (Fig. 1d). The pattern of response changed markedly at low nitrogen supply with both species showing a significant (1.7 fold) increase in C:N ratio. In response to temperature, the slow-growing *B. erectus* showed a more mixed response with a marked increase (60%) at 30 °C and a decline (10%) at 7 °C*.* The fast-growing *B. hordeaceus* had higher ratios overall with a steady decline with a rise in temperature.

**Table 2:** Physiological response

Parameter	N-treatment	Temp (°C)	*Bromus erectus*	*Bromus hordeaceus*	Test statistics		
					t	df	p
Net photosynthesis (nmol/g s)	FA	7	19.49 ± 2.7	38.90 ± 5.3	*5.6*	*3*	*0.01*
		20	268.13 ± 13.1	518.51 ± 42.8	*9.6*	*2*	*0.01*
		30	183.35 ± 71.5	404.52 ± 45.73	*8.2*	*2*	*0.01*
	LA	7	11.12 ± 4.0	17.26 ± 7.8	1.2	3	0.31
		20	186.39 ± 71.5	296.11 ± 19.84	2.5	2	0.12
		30	54.93 ± 11.1	25.54 ± 1.4	*4.5*	*2*	*0.02*
Respiration in the dark (nmol/g s)	FA	7	6.42 ± 1.2	16.68 ± 6.5	2.7	2	0.11
		20	99.66 ± 18.4	183.74 ± 53.3	2.5	2	0.12
		30	102.43 ± 3.3	221.69 ± 14.35	*4.5*	*2*	*0.04*
	LA	7	6.12 ± 1.2	6.81 ± 1.24	0.6	4	0.53
		20	66.03 ± 16.4	90.40 ± 25.4	1.3	3	0.25

Rates of net photosynthesis and leaf respiration in the dark under their respective growth conditions (ex: plants that where grown at 20 °C were measured at 20 °C, plant that were grown at 30 °C were measured at 30 °C)

Values are means of n = 3 samples and shown with standard deviation. Test statistics show results from T-test between the species

### Nitrogen use efficiency

Under FA conditions, both species *B. erectus* and *B. hordeaceus* showed a similar pattern of response, being minimal at 20 °C (p = 0.003, Fig. 2b). NUE rates were higher but similar, at 7 °C and 30 °C with the fast-growing *B. hordeaceus* always having lower values, 12% at 7 °C and 22% at 30 °C (p = 0.007 and p = 0.000, respectively). Responses of NUE to low nitrogen availability were species-specific (Table 1). In the slow-growing *B. erectus* NUE increased slightly from 7 °C to 20 °C, and then markedly at 30 °C, resulting in a 59% higher NUE when compared to FA conditions. In contrast, the fast-growing *B. hordeaceus* had similar NUE at 7 °C and 20 °C, which then declined to only 208.4 g/g at 30 °C.

### Leaf carbon uptake and release

For both species, the uptake rates were highest when grown at 20 °C with those for the fast-growing *B. hordeaceus* being twice as high as those for the slow-growing *B. erectus* (Fig. 2a). Both chilling and warming reduced these rates, with NP_max_ close to zero at 7 °C for both species but a smaller decline at 30 °C. Under LA conditions the response pattern for both species remained similar to that under FA conditions (Fig. 2a) but rates at 20 °C and 30 °C were lower than under FA.

Leaf respiration rates in the dark were highest at 30 °C growth temperature with those of the fast-growing *B. hordeaceus* being twice as those of the slow-growing *B. erectus* (Table 2). At LA conditions, the dark respiration rates had a maximum at 20 °C.

### Optimal temperature for photosynthesis (T_opt_)

**Fig. 3:**
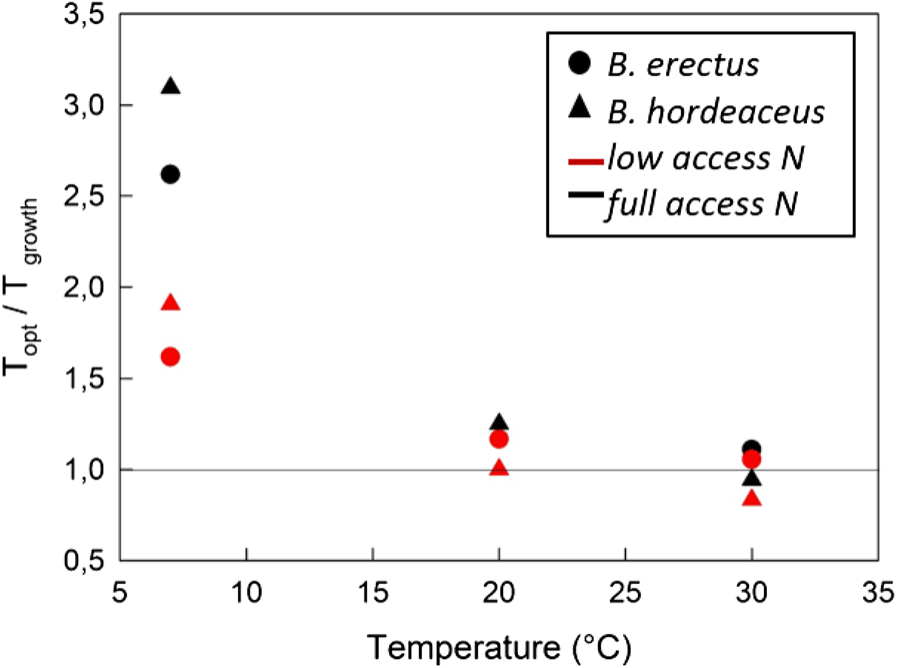
Optimal growth temperature. Displayed is a ratio between the optimal temperature for photosynthesis (T_opt_) and the growth temperature during the experiment. These values are related to the growth temperature to visualize deviations and acclimation. A ratio of one indicates that the optimal temperature matches the growth temperature, values above one indicate that the optimal temperature was higher than the growth temperature and vice versa

Under FA conditions both species showed very similar responses in the temperature for optimal net photosynthesis, T_opt_ (Fig. 3). T_opt_ was almost identical to growth temperature at 20 °C and 30 °C indicating that acclimation to these warmer growth temperatures was realised. However, acclimation was less obvious at 7 °C with T_opt_ being more than twice as high as the growth temperature. Under low nitrogen delivery, this pattern remained similar for both species with the exception that T_opt_ was closer to the actual growth temperature at 7 °C.

### Daily carbon budget

**Fig. 4:**
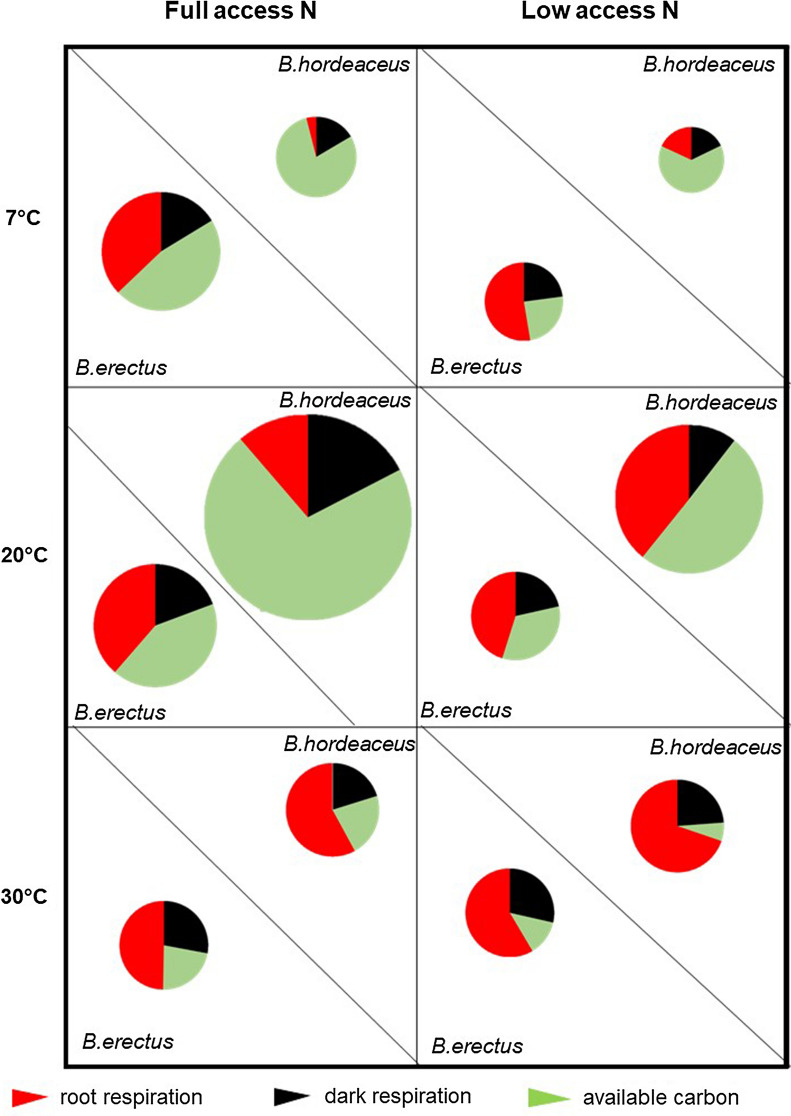
Daily carbon budget. Dry weight-based rates for net photosynthesis, dark respiration and root respiration rates were converted to a per day basis and the daily rates were weighted according to the number of hours that each gas exchange parameter took place (NP:16 h; DR: 8 h and RR: 24 h). The total size of the circle reflects the RGR that is normalized for fast-growing *B.hordaceus* at control conditions to 100%

Under FA and at 20 °C, the fast-growing *B. hordeaceus* lost only one-fourth of its daily available carbon via root and leaf dark respiration, while this was more than a half for the slow-growing *B.erectus* (Fig. 4). At 7 °C, this pattern remained almost identical in both species, but the total amount of available carbon (size of the circle) was much reduced in the fast-growing *B. hordeaceus*, but remained almost identical for the slow-growing *B. erectus*. At 30 °C total available carbon decreased and because total respiration losses increased in both species the available carbon fraction was cut to one-fourth (Fig. 4).

Under LA conditions total available carbon for the slow-growing *B. erectus* was almost constant at all temperatures with totals being lower than at FA at 7 °C and 20 °C but almost identical at 30 °C (Fig. 4). In contrast, total available carbon for the fast-growing *B. hordeaceus* was highest at 20 °C, almost twice that at the other temperatures, with the total being lower than under FA at 7 °C and 20 °C but, as for the slow-growing *B. erectus*, almost identical at 30 °C. The proportion of total available carbon allocated to root respiration was higher than in FA which lowered the daily available carbon fraction in both species, but much more so in the fast-growing *B. hordeaceu*s. At 30 °C, carbon losses, due to increased root and dark respiration, were maximal in both species and resulted in the smallest available carbon fractions (Fig. 4).

## Discussion

Our results indicate clear differences in trait coordination between a slow growing grass perennial and a fast growing, invasive competitor. Any fast acclimation to growth conditions via ecophysiological trait adjustments is costly, especially when low metabolic rates associated with low growth temperatures occur in combination with nutrient depletion. By having a set of well-conserved adaptive functional traits, slow-growing perennials might have benefits when handling such a combination of stressors.

### A slow grower’s response to changes in growth temperature, nutrient limitation and their combination

Plant traits are strongly correlated with temperature [39] and at 7 °C *B. erectus* had almost identical RGR as at 20 °C (control conditions) due to a well-balanced adjustment of its functional traits. Because NAR and SLA are co-dependent in the calculation of RGR [28], for RGR to remain at levels similar to control conditions, the significant decrease in SLA involved a simultaneous increase in NAR (more than double to that of control conditions (Fig. 1c)). This indicates that, at low temperature, the net dry weight gain was converted to higher investments in leaf stability, making them thicker and more sustainable and agrees with the common response of plants when grown at low temperatures [32, 61]. Surprisingly, growing in the cold also resulted in higher investments in root tissue, although root growth is usually known to be limited at temperatures ~ 6 °C (Alvarez‐Uria and Körner [2]). These results could be explained via a the following response cascade starting with the carbon uptake in the leaf: At 7 °C, due to a lack of thermal acclimation of net photosynthesis (Fig. 3), carbon uptake rates were much reduced (Fig. 2a, Table 2). As a response to operating at such low, sub-optimal uptake rates, NUE was increased to enhance the efficiency of carbon incorporation into biomass (Fig. 2b). For the plant's growth economics, this is a costly process, which was displayed in a higher C:N ratio. The higher C:N ratio in the leaves can then be the trigger for the plant to allocate biomass investments towards the root in order to increase the surface tissue for nutrient uptake according to the “optimal resource partitioning strategy”. According to this model, plants respond to environmental factors that limit the acquisition of below-ground resources relative to above-ground resources by shifting their partitioning to tissues associated to gaining the relatively limiting resource [5].

In contrast to the cold temperature response, the RGR of *B.erectus* was reduced when growing at higher temperatures (30 °C) (Fig. 1a). This occurs because NAR remained at the same level as at the 20 °C growth temperature but SLA decreased, which is a typical response of plants to warming [23]. In comparison to growing at 20 °C, the carbon uptake rates were reduced (Fig. 2a, Table 2), while simultaneously, respiration rates increased (Table 2). Temperature is known to have a crucial influence on respiratory CO_2_ efflux [3] and therefore the daily carbon budget of a plant [32]. As a result, the carbon losses were so high that the available carbon fraction for growth was cut to one-fourth (Fig. 4) making it impossible for NAR to increase.

Limited nitrogen delivery changed the response patterns and the previously described reaction norm of the slow growing *B.erectus* in response to temperature*.* Now, RGR was generally reduced to around 65% of FA conditions and was even lower at both treatment temperatures (7 °C and 30 °C). The cold + low nitrogen treatment could be identified as the most limiting for *B.erectus,* despite having adjusted so well under FA conditions. Neither the up-regulation of NAR, nor the down regulation of SLA demonstrated under FA conditions was realised, resulting in lower RGR most possibly linked to a limited potential to up regulate NUE (Fig. 2b) under LA conditions. In order to compensate for the lower nutrient availability, the close functional coordination between root and shoot traits [59] resulted in higher investments in root biomass (accompanied with higher root respiration losses) indicating that leaf-acquired resources are linked to the root economics and vice versa.

In direct contrast, when *B. erectus* was exposed to warming (30 °C) in combination with LA conditions, the growth was less affected and only slightly lower than under FA conditions. However, due to increased root and leaf dark respiration rates the amount of available carbon resources were at their lowest (Fig. 4), and, to improve the efficiency of carbon incorporation into biomass, NUE was highest. As found at 7 °C under FA conditions, this process appears to be costly and results in high C:N ratios and also the highest R:S ratio, indicating a coordinated variation of root and shoot nitrogen content as well as SLA [11, 12]. For *B. erectus*, this could reflect the species’ habitat specialisation, as *B. erectus’* favoured habitat types are warm–nutrient-poor environments such as calcareous grasslands of the Mesobromion alliance [17], of which it is a characteristic, name-giving species. In general, species with a slow-growing traits syndrome are more successful under low nutrient conditions [11, 12, 27, 34].

### What is different in the fast-grower?

In contrast to *B. erectus*, *B. hordeaceus* had a fast-grower’s life strategy and grew twice as fast, had doubled NP_max_ and significantly higher SLA at 20 °C with FA conditions. This also displays the invasive character of *B. hordeaceus*, because invasive alien species are known to have higher values for performance-related traits than non‐invasive species [56]. The following section highlights the differences in the reaction norm to changes in growth temperature, nutrient supply and their combinations for *B. hordeaceus* in comparison to the slow-growing *B. erectus*.

The main difference was that *B. hordeaceus* had a sharp maximum in RGR at 20 °C and could not shift to grow well at higher or lower temperatures. Although NAR, in general, was about 30% higher in *B. hordeaceus* than in *B. erectus,* the impossibility to adjust NAR in response to temperature resulted in a drastic decrease in RGR. When exposed to warm growing temperatures RGR was then similar to those of the slow growing *B. erectus* and when exposed to cold temperatures RGR were even below those of *B. erectus*.

‘Fast’ traits, are costly in the face of any kind of resource shortfall [48], and fast-growing species are less tolerant to any changes in resource availability (whether water, nutrients or light). It has been shown previously, that the relatively low rates of root respiration in fast-growing grasses in comparison to slow growing ones are a result of the lower costs for nutrient uptake [52]. Thus, it is not surprising to find that LA conditions had more severe effects the total carbon budget of *B. hordeaceus* than of *B. erectus*. Especially when exposed to a combination of cold growing temperatures and restricted nitrogen supply, the inability to adjust SLA and NUE (which was the response of *B. erectus* leading to only 10% reduction of RGR) results in the lowest RGR, which is less than 50% from LA 20 °C levels and even below that of *B. erectus*. Such a strongly limited growth performance was also shown when nitrogen limitation occurred in combination with warmer growth temperature; conditions that did not greatly affect RGR of *B. erectus* (Fig. 1a). Here, for *B. hordeaceus* RGR was almost halved together with a reduction of NUE and an increase in R:S of less significance than in *B. erectus*. Effects on the daily available carbon fraction were additive, such that the uptake rates were reduced by temperature plus the carbon losses were higher because of increased root respiration due LA conditions (Fig. 4). From the plant carbon economics perspective, this meant that any resource allocation as a response to environmental changes became problematic, simply due to the fact that available resources were low.

## Conclusions

We can support our initial hypothesis that nitrogen limitation will limit growth performance independent of growth strategy because we saw that the 50% reduction of N-availability at optimal temperature decreased RGR to the same amount in both species. However, both species seem to use the same mechanism to achieve this (increasing NUE). A good nutrient acquisition capacity could be the result of low biomass density at least in the fast-growing grasses [50]. As an additional explanation it needs to be considered that although the nitrogen availability was reduced to 50% in the growth units, the nutrient availability was still higher than it would be within a nitrogen depletion scenario in natural conditions (such as found in a limestone grassland, [26]. However, for our approach, the decision to reduce N by 50% displayed an experience-based trade-off between experimental handling (overall time of the plants in the growth units) and effect size, which was proven to be significant in most treatments.

Temperature affects leaf energy balance [20, 29], metabolic rate [19] and plant growth rate [32], and many ecological traits are known to be correlated with temperature [45], Went 1953), including leaf nutrient content or leaf mass per unit area and leaf lifespan [47, 62]. On a global basis, mean annual temperature has been shown to strongly correlate with plant traits [39] but slow‐ and fast‐growing species did not appear to differ in their plasticity of RGR in response to growth temperature [32]. Therefore, our second hypothesis handled the response of plant ecophysiological traits to changes in temperature and we were suggesting this to be independent of growth strategy (fast vs. slow). Our results support this hypothesis by showing that significant increases in carbon losses via both shoot and root respiration, a reduction of the root biomass and inflexible NAR values, were similar responses to changes in temperature in both species. Most prominent, in response to chilling, the unaltered carbon budget pattern as a result of a uniform reduction of the absolute rates carbon uptake and release rates (Fig. 4), was uniform across species and growth strategy. Nevertheless, the effect size of these responses was consistently higher in the fast-growing *B. hordeaceus* than it was in the slow-growing *B. erectus* and this led to more drastic effects in RGR reduction in the fast-growing species. Accordingly, the fast-growing *B. hordeaceus* showed a marked optimum at 20 °C growth temperature whilst *B. erectus* did not.

Finally, we posed the question of whether potential benefits of a more conservative life strategy in slow-growing plants through functional traits vanish when ecophysiological trait coordination is needed as well, in a combined stress scenario. Our results imply, that for the fast-growing invader *B. hordeaceus* a reduction of nitrogen availability in combination with a temperature increase may indeed lead to a disadvantage in comparison to the slow-growing perennial species *B.erectus* and this may explain why nutrient-rich habitats often experience more invasion than resource-poor habitats [6, 14]*.* However, the absolute values of traits (such as RGR, SLA or NP_max_) were similar between fast-and slow-growing species when the plants where grown at suboptimal temperature and at low- nitrogen availability. This implies that any differentiation between the two growth strategies becomes difficult in such a scenario and a growth strategy convergence can occur as a result from combined stress effects.

## Methods

### Species selection

This research was concentrated on graminoids because, not only were they suitable for the experimental conditions (growth units) but they are also the dominant vegetation in many habitats, including grassland, salt-marsh, reed swamp and steppes and include some of the most versatile plant functional types. We selected two C_3_ grass species, *Bromus erectus* and *Bromus hordeaceus*.

*B. hordeaceus* is a grass species native to Europe. It has several features shared by successful invasive species including a short life cycle and a predominantly autogamous breeding system (CABI [7]). It is an annual species of grass (Poaceae) and flowers from May until July [8]. *B. hordeaceus* has been introduced into parts of North and South America and Australia. It is a weed of crop fields, grasslands, orchards and turf where it competes with native vegetation and monopolizes resources. The species is described to be fast growing and to have a very low LMA (LMA = 20 g/m^2^) even in comparison to other fast-growing species [57].

*B. erectus* is a grass species also native to Europe. It is a medium-tall grass which forms loose tussocks and produces few tillers. It is a perennial grass that flowers in May/June [8]. This species is mainly found on warm, well-drained, calcareous soils in upland areas. The species is described to have a resource-acquisition strategy as a slow growing species (LMA = 60.54 g/m^2^, [42]).

### Experimental design

Seedlings were germinated on a mixture of sand and vermiculite (1:1). Immediately after the appearance of the second leaf, seedlings were removed from the sand, washed carefully and placed into custom-made aeroponic growth units (Biotronic AB, Sweden) that allow accurate control over the nutrient supply to the plants [25]. One growth unit contained up to 84 seedlings. Four growth units (2 for each species) were placed in a growth cabinet with a selected constant temperature, 16-h light period (7 am–11 pm) with a light intensity of 200 μmol photons/m^2^ s and 70% relative humidity. The temperature of the nutrient solution was kept the same as the air temperature, with a maximum deviation of ± 1 °C. After transplantation, plants were first acclimated to the growing conditions in the aeroponic units, and this was determined to be completed once the pH and the conductivity of the medium solution became stable and the nutrient uptake had recovered as assessed by regular nutrient titrations being necessary (pH 5.5, conductivity 99–101 µS in 6 dm^3^ solution).

The experiment consisted of 6 treatments for each species, in a 3 × 2 factorial design: three temperature conditions (7 °C (chilling), 20 °C (control) and 30 °C (warming)) and two nutrient conditions (free access and low access (50% reduced) nitrogen). Plants incubated at 20 °C were considered the control group because this temperature has been described to be optimal for grasses from steppe or meadow vegetation in Europe [30]. Under free access nitrogen (FA) treatments, the seedlings were supplied with mineral nutrients in a proportion that was known to be optimal for growth [24], with a nitrogen concentration that was low, but optimal (30 ± 2 mg in 6 dm^3^ solution, [25]. In the nitrogen limited (LA) treatments, the proportion of N in the stock solutions was reduced by 80% and nitrogen supply was adjusted manually on a daily basis using a 1 molar ammonium-nitrate stock solution, so that the N supply (as mg N/day) was reduced to 50% of that required by the seedlings for optimal growth. The supply of all the other nutrients remained unchanged.

### Growth analysis

Harvests for the growth analysis were performed at regular intervals each with five replicates of each species. The plants were divided into leaves and roots. Fresh and dry mass (after 4 days in the dry oven at 80 °C) were recorded. Leaf area was measured using a LI-COR Li-3000C leaf area meter (LI-COR Inc., Lincoln, NE, USA). The dry material was ground to a fine powder using a mortar and pestle and analyzed for total C and N concentration by mass spectrometry (Isotope ratio mass spectrometer (DeltaV, Thermo Fisher Scientific, Bremen, Germany; Elemental analyzer (Flash EA 2000, Thermo Fisher Scientific, Bremen, Germany)) as described by Werner et al. [58]. The C:N ratio as well as the NUE (increase in dry weight per unit of nitrogen) was calculated. RGR was calculated according to Lambers and Poorter [28]:

$$ {\text{RGR }} = {\text{ LAR}}\; \times \;{\text{NAR}}  $$

LAR (leaf area ratio) is the product of SLA, and the leaf weight ratio (LWR, fraction of biomass allocated to the leaves). Net assimilation rate (NAR) is defined as the rate of increase in plant weight per unit leaf area.

Additional growth-related parameters such as LMA and the R:S ratio were determined.

### Resource uptake, partitioning and allocation

CO_2_ exchange was measured in order to demonstrate changes in the carbon exchange of the plants. Therefore, as soon as the third leaf was fully expanded (indicated by stable leaf dry weight) three replicates were harvested and experiments performed. This approach was chosen to assure sampling of plants at similar physiological stages of development and that the sampling material (the third leaf) was completely developed under treatment conditions. The whole plant was harvested carefully and with the roots immersed in a small plastic container containing the original nutrient solution. The third leaf was carefully positioned in the gas exchange cuvette (3010-GWK1, Walz, Effeltrich, Germany). The cuvette was attached to an infrared gas analyser (LI-COR 6400, LI-COR Inc., Lincoln, NE, USA) to measure the CO_2_ fluxes. CO_2_ concentration in the cuvette was adjusted to saturating conditions (1000 ppm) and the light was set to 200 µmol photons/m^2^ s (mimicking the conditions in the growth chamber). The temperature was set to mimic the growth conditions for an initial equilibration phase. Once the signal was stable, net photosynthesis (NP, light in the cuvette switched on), and dark respiration (light switched off and cuvette completely shaded) were measured at different temperatures (5, 7, 10, 15, 20, 25, 30, 35 and 40 °C, in a randomly chosen sequence). From these measurements, we obtained maximum net photosynthesis (NP_max_), optimal temperature for photosynthesis (T_opt,_ the temperature range over which net photosynthesis was above 90% of its maximum), net photosynthesis and leaf respiration in the dark (DR) at the ambient growth temperature. Thermal acclimation was displayed as a ratio between T_opt_ and the growth temperature during the experiment. A ratio of one indicated that the optimal temperature matches the growth temperature, values above one indicate that the optimal temperature was higher than the growth temperature and vice versa. Carbon uptake rates were expressed on a dry weight basis, because the higher assimilation rates of fast-growing species on an area basis, are ‘diluted’ by having a higher SLA simultaneously [15, 40, 43] and the aim of this study was to compare the net carbon gain rather than the net photosynthetic rate. It was stated, therefore, that comparisons of photosynthesis and growth can only be made per unit plant weight and per unit of time [44].

Once the leaf gas exchange measurement was finished, the roots were cut off and root respiration (RR) was determined as the decrease of O_2_ concentration in a liquid phase oxygen electrode system (CB1D, Hansatech Instruments, Norfolk, United Kingdom). Measurements were made at 7 °C, 20 °C, and 30 °C.

An estimate of each individual plant's daily carbon budget was obtained by converting net photosynthesis, respiration (in the dark) and root respiration rates to a per day basis according to the number of hours per day that each gas exchange parameter took place (NP 16 h, DR 8 h and RR 24 h). In order to reflect the whole plant budget, these rates were weighted according to the plants R:S ratio.

### Statistics

All statistical analyses were performed using SPSS software (SPSS statistics 24, IBM Analytics, New York). Prior analysis, the within-group normal distribution was checked using Shapiro–Wilk tests.

To evaluate the effects of temperature, nitrogen availability and their interaction with the dependent variables SLA, NP_max_, NUE and C:N ratio, we applied a univariate linear model. Species (2 discrete levels: *B. erectus* vs*. B. hordeaceus*), temperature (3 discrete levels: 7 °C, 20 °C and 30 °C) and nitrogen treatment (2 discrete levels: full access vs. low access) were treated as discrete explanatory variables. We evaluated single effects as well as all two-way and three-way interactions. T-tests were performed to compare between the species on selected groups.

## Data Availability

The datasets used and/or analysed during the current study are available from the corresponding author on reasonable request. All authors read and approved the final manuscript.

## Abbreviations

SLA: Specific leaf area
RGR: Relative growth rate
LMA: Leaf mass area
R:S: Root to shoot ratio
C:N: Carbon to nitrogen ratio
NUE: Nitrogen use efficiency
R: Respiration
NP: Net photosynthesis
NP_max_: Maximum net photosynthesis
FA: Free access nitrogen treatment
LA: Low access nitrogen treatment
NAR: Net assimilation rate
T_opt_: Optimal temperature for photosynthesis
LAR: Leaf area ratio
LWR: Leaf weight ratio
RR: Root respiration
DR: Dark respiration

## References

[1] Aerts R, Chapin FS (2000). The mineral nutrition of wild plants revisited: a re-evaluation of processes and patterns. Adv Ecol Res.

[2] Alvarez-Uria P, Körner C (2007). Low temperature limits of root growth in deciduous and evergreen temperate tree species. Funct Ecol.

[3] Atkin OK, Bruhn D, Hurry VM, Tjoelker MG (2005). Evans Review No 2: The hot and the cold: unravelling the variable response of plant respiration to temperature. Funct Plant Biol.

[4] Baltzer JL, Thomas SC (2007). Physiological and morphological correlates of whole plant light compensation point in temperate deciduous tree seedlings. Oecologia.

[5] Bloom AJ, Chapin FS, Mooney HA (1985). Resource limitation in plants-an economic analogy. Annu Rev Ecol Syst.

[6] Burke MJW, Grime JP (1996). An experimental study of plant community invasibility. Ecology.

[7] CABI *Bromus hordeaceus* In: Invasive Species Compendium Wallingford UK: CAB International. 2018. https://www.cabi.org/isc.

[8] Clayton WD, Vorontsova MS, Harman KT, Williamson H. GrassBase–The Online World Grass Flora. 2006. http://www.keworg/data/grasses-dbhtml.

[9] Cornelissen JHC, Lavorel S, Garnier E, Diaz S, Buchmann N, Gurvich DE, Pausas JG (2003). A handbook of protocols for standardised and easy measurement of plant functional traits worldwide. Aust J Bot.

[10] Craine J, Tilman DG, Wedin DA, Reich P, Tjoelker MG, Knops JMH (2002). Functional traits productivity and effects on nitrogen cycling of 33 grassland species. Funct Ecol.

[11] Craine JM, Lee WG (2003). Covariation in leaf and root traits for native and non-native grasses along an altitudinal gradient in New Zealand. Oecologia.

[12] Craine JM, Lee WG, Bond WJ, Williams RJ, Johnson LC (2005). Environmental constraints on a global relationship among leaf and root traits of grasses. Ecology.

[13] Cronk QC, Fuller JL. Plant invaders: the threat to natural ecosystems. People and Plant Conservation Manuals. 2^nd^ edn. Earthscan, Routledge, New York; 2013.

[14] Daehler CC (2003). Performance comparisons of co-occurring native and alien invasive plants: implications for conservation and restoration. Annu Rev Ecol Evol Syst.

[15] Dijkstra P, Lambers H (1989). A physiological analysis of genetic variation in relative growth rate within *Plantago major* L. Funct Ecol.

[16] Enrique G, Olmo M, Poorter H, Ubera JL, Villar R (2016). Leaf mass per area (LMA) and its relationship with leaf structure and anatomy in 34 Mediterranean woody species along a water availability gradient. PLoS ONE.

[17] Ellenberg H (1996). Vegetation Mitteleuropas mit den Alpen.

[18] Funk JL, Vitousek PM (2007). Resource-use efficiency and plant invasion in low-resource systems. Nature.

[19] Gillooly JF, Brown JH, West GB, Savage VM, Charnov EL (2001). Effects of size and temperature on metabolic rate. Science.

[20] Harrison SP, Prentice IC, Barboni D, Kohfeld KE, Ni J, Sutra JP (2010). Ecophysiological and bioclimatic foundations for a global plant functional classification. J Veg Sci.

[21] Hodgson JG, Wilson PJ, Hunt R, Grime JP, Thompson K (1999). Allocating C-S-R plant functional types: a soft approach to a hard problem. Oikos.

[22] Holste EK, Kobe RK, Vriesendorp CF (2011). Seedling growth responses to soil resources in the understory of a wet tropical forest. Ecology.

[23] Hudson JMG, Henry GHR, Cornwell WK (2011). Taller and larger: shifts in Arctic tundra leaf traits after 16 years of experimental warming. Glob Change Biol.

[24] Ingestad T (1971). A definition of optimum nutrient requirements II. Physiol Plantarum.

[25] Ingestad T, Lund AB (1979). Nitrogen stress in birch seedlings I. Growth Technique and Growth. Physiol Plantarum.

[26] Köhler B, Ryser P, Güsewell S, Gigon A (2001). Nutrient availability and limitation in traditionally mown and in abandoned limestone grasslands: a bioassay experiment. Plant Soil.

[27] Körner C. Plant‐environment interactions In: Strasburger's Plant Sciences; Springer, Heidelberg; 2013. pp 1065–1166

[28] Lambers H, Poorter H (2004). Inherent variation in growth rate between higher plants: a search for physiological causes and ecological consequences. Adv Ecol Res.

[29] Lambers H. Leaf energy budgets: Effects of radiation and temperature In: H Lambers FS Chapin and TL Pons (Eds) Plant Physiological Ecology. New York: Springer; 1998

[30] Larcher W (2004). Physiological Plant Ecology: Ecophysiology and Stress Physiology of Functional Groups.

[31] Lavorel S, Garnier E (2002). Predicting changes in community composition and ecosystem functioning from plant traits: revisiting the Holy Grail. Funct Ecol.

[32] Loveys BR, Scheurwater I, Pons TL, Fitter AH, Atkin OK (2002). Growth temperature influences the underlying components of relative growth rate: an investigation using inherently fast-and slow-growing plant species. Plant Cell Environ.

[33] Lusk CH, Jorgensen MA (2013). The whole-plant compensation point as a measure of juvenile tree light requirements. Funct Ecol.

[34] Mason NWH, Richardson SJ, Peltzer DA, de Bello F, Wardle DA, Allen RB (2012). Changes in coexistence mechanisms along a long-term soil chronosequence revealed by functional trait diversity. J Ecol.

[35] Meinzer FC, Campanello PI, Domec JC, Gatti MG, Goldstein G, Villalobos-Vega R (2008). Constraints on physiological function associated with branch architecture and wood density in tropical forest trees. Tree Physiol..

[36] Meinzer FC, Woodruff DR, Domec J-C, Goldstein G, Campanello PI, Gatti MG (2008). Coordination of leaf and stem water transport properties in tropical forest trees. Oecologia.

[37] Meinzer FC, McCulloh KA, Lachenbruch B, Woodruff DR, Johnson DM (2010). The blind men and the elephant: the impact of context and scale in evaluating conflicts between plant hydraulic safety and efficiency. Oecologia.

[38] Mueller KE, LeCain DR, McCormack ML, Pendall E, Carlson M, Blumenthal DM (2018). Root responses to elevated CO_2_ warming and irrigation in a semiarid grassland: integrating biomass length and lifespan in a 5-year field experiment. J Ecol.

[39] Moles AT, Perkins SE, Laffan SW, Flores-Moreno H, Awasthy M, Tindall ML, Anand M (2014). Which is a better predictor of plant traits: temperature or precipitation?. J Veg Sci.

[40] Mooney HA, Ferrar PJ, Slatyer RO (1978). Photosynthetic capacity and carbon allocation patterns in diverse growth forms of Eucalyptus. Oecologia.

[41] Ordoñez JC, Bodegom PM, Witte J-PM, Wright IJ, Reich PB, Aerts R (2009). A global study of relationships between leaf traits climate and soil measures of nutrient fertility. Global Ecol Biogeogr..

[42] Pérez-Ramos IM, Volaire F, Fattet M, Blanchard A, Roumet C (2013). Tradeoffs between functional strategies for resource-use and drought-survival in Mediterranean rangeland species. Environ Exp Bot.

[43] Poorter H, Lambers H (1989). Interspecific variation in relative growth rate: on ecological causes and physiological consequences. Causes and consequences of variation in growth rate and productivity of higher plants.

[44] Poorter H, Remkes C, Lambers H (1990). Carbon and nitrogen economy of 24 wild species differing in relative growth rate. Plant Physiol.

[45] Rawson H (1992). Plant responses to temperature under conditions of elevated CO_2_. Aust J Bot.

[46] Reich PB, Ellsworth DS, Walters MB (1998). Leaf structure (specific leaf area) modulates photosynthesis–nitrogen relations: evidence from within and across species and functional groups. Funct Ecol.

[47] Reich PB, Oleksyn J (2004). Global patterns of plant leaf N and P in relation to temperature and latitude. P Natl Acad Sci USA.

[48] Reich PB (2014). The world-wide ‘fast–slow’ plant economics spectrum: a traits manifesto. J Ecol.

[49] Russo SE, Davies SJ, King DA, Tan S (2005). Soil-related performance variation and distributions of tree species in a Bornean rain forest. J Ecol.

[50] Ryser P, Lambers H (1995). Root and leaf attributes accounting for the performance of fast-and slow-growing grasses at different nutrient supply. Plant Soil.

[51] Saud S, Fahad S, Cui G, Yajun C, Anwar S (2020). Determining nitrogen isotopes discrimination under drought stress on enzymatic activities, nitrogen isotope abundance and water contents of Kentucky bluegrass. Sci Rep.

[52] Scheurwater I, Cornelissen C, Dictus F, Welschen R, Lambers H (1998). Why do fast- and slow-growing grass species differ so little in their rate of root respiration considering the large differences in rate of growth and ion uptake?. Plant Cell Environ.

[53] Suzuki N, Rivero RM, Shulaev V, Blumwald E, Mittler R (2014). Abiotic and biotic stress combinations. New Phytol.

[54] Tilman D, Wedin D (1991). Plant traits and resource reduction for five grasses growing on a nitrogen gradient. Ecology.

[55] Tjoelker MG, Craine JM, Wedin D, Reich PB, Tilman D (2005). Linking leaf and root trait syndromes among 39 grassland and savannah species. New Phytol.

[56] Van Kleunen M, Weber E, Fischer M (2010). A meta-analysis of trait differences between invasive and non-invasive plant species. Ecol Lett.

[57] Waddell HA, Simpson RJ, Lambers H, Henderson B, Ryan MH, Garden DL, Richardson AE (2016). Phosphorus-utilisation efficiency and leaf-morphology traits of Rytidosperma species (wallaby grasses) that differ in their growth response to phosphorus fertilisation. Aust J Bot.

[58] Werner RA, Bruch BA, Brand WA (1999). ConFlo III-an interface for high precision d13C and d15N analysis with an extended dynamic range. Rapid Commun Mass Spectrometry.

[59] Westoby M, Wright IJ (2006). Land-plant ecology on the basis of functional traits. Trends Ecol Evol.

[60] Wilson PJ, Thompson KEN, Hodgson JG (1999). Specific leaf area and leaf dry matter content as alternative predictors of plant strategies. The New Phytol.

[61] Wolfe DW (1991). Low temperature effects on early vegetative growth leaf gas exchange and water potential of chilling-sensitive and chilling-tolerant crop species. Ann Bot.

[62] Wright IJ, Reich PB, Cornelissen JHC, Falster DS, Groom PK, Hikosaka K (2005). Modulation of leaf economic traits and trait relationships by climate. Global Ecol Biogeogr.

